# Lung Abscess: An Early Complication of Lung Transplantation in a Patient with Cystic Fibrosis

**Published:** 2017-11-01

**Authors:** I. Markelić, M. Jakopović, W. Klepetko, F. Džubur, A. Hećimović, M. J. Makek, M. Samaržija, A. V. Dugac

**Affiliations:** 1 University Hospital Centre Zagreb, Department for Respiratory Diseases, Zagreb, Croatia; 2 School of Medicine, University of Zagreb, Croatia; 3 Vienna General Hospital, Department of Surgery, Division of Thoracic Surgery, Waehringer Guertel 18-20, Vienna A-1090, Austria; 4 Medical University of Vienna, Austria

**Keywords:** Lung transplantation, Lung abscess, Cystic fibrosis, *Acinetobacter baumannii*

## Abstract

A 22-year-old woman with cystic fibrosis (CF) developed lung abscess, as a rare complication caused by multidrug-resistant (MDR) *Acinetobacter baumannii* infection, after lung transplantation (LT). After 6 months of long-term antibiotic therapy, the abscess was successfully eliminated. In reviewed published literature, no previous report was found describing this kind of complication caused by MDR *A. baumannii* in post-LT patient with CF. In our experience, lung abscess in LT recipients with CF can be successfully treated with prolonged antibiotic therapy.

## INTRODUCTION

Lung transplant (LT) recipients are at increased risk for both community-acquired and nosocomial pathogens, which may develop at various time points. Bacterial infection is one of the most serious complications in LT recipients and a significant determinant of the transplant outcome, with bacterial pneumonias being the leading cause in this category. However, lung abscess is an extremely rare complication according to recent medical literature. Herein, we report on a case of multidrug-resistant (MDR) *Acinetobacter baumannii* and *Pseudomonas aeruginosa *infection presenting with lung abscess in a LT recipient with cystic fibrosis (CF). 

## CASE REPORT

A 22-year-old woman diagnosed with CF at the age of three years entered end-stage lung disease in 2011, aged 16. She had frequently been admitted to hospital due to lung disease exacerbations and treated with various antibiotics leading to rapid decrease in her FEV_1_ (30% from a baseline of 64%). Three years later, in January 2014, she was registered for LT program in Adult Cystic Fibrosis Centre. While waiting for the LT, in July 2014 the patient presented with right-sided pneumothorax, which recurred four months later. In between, she had been admitted to hospital many times due to exacerbations of her CF. Her lung function continued to decrease rapidly reaching an FEV_1_ of only 19.7%.

A double LT under ECMO support was performed on January 17, 2015 in AKH Wien. The postoperative course was prolonged due to a reperfusion edema with the need for continuous ECMO support and complicated with MDR *A. baumannii* and *P. aeruginosa* infections. Computed tomography (CT) of the chest revealed two abscesses on the left lung, one in pleural cavity and another in the lung parenchyma.

**Figure 1:**
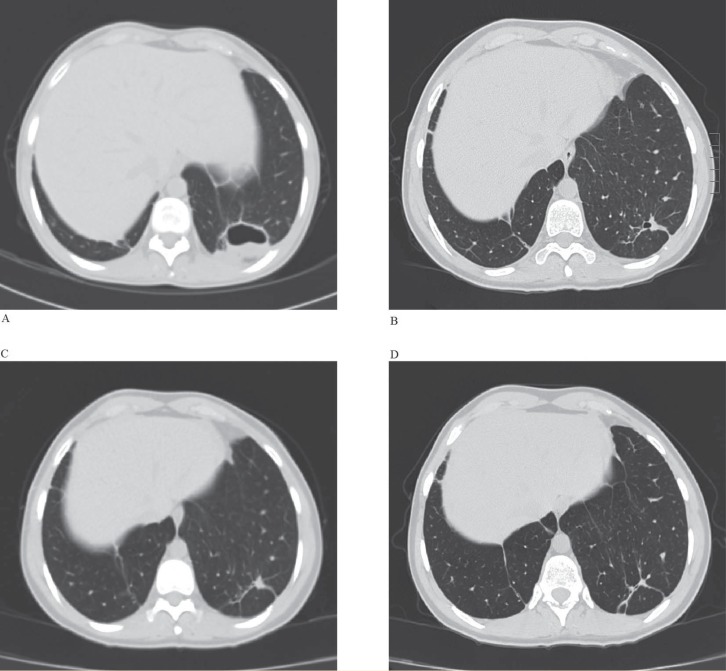
CT scans of the lung abscess regression over the treatment period. A) Lung abscess of the left lung after two months of beginning of the treatment (4/2015). B) Beginning of abscess resolution (7/2015). C) Complete resolution of the abscess (10/2015). D) Intrathoracic status a year after the treatment (3/2016

The lung abscess was drained with a pigtail catheter and antibiotic therapy was initiated—colistin and piperacillin/tazobactam intravenously (iv) with tobramycin inhalations. The treatment led to significant reduction of the abscess cavity so after 10 days the pigtail catheter was removed. On March 26, 2015 she was transferred to our Department where she remained until April 24, 2015 (Fig 1A) continuing the above-mentioned antibiotics treatment along with frequent microbiological surveillance. She participated in our pulmonary rehabilitation program. By the end of the hospital stay her lung function was inclining. After 30 days, iv antibiotic treatment was replaced with colistin and tobramycin inhalations. Regression in lung abscess dimensions was observed (Fig 1B). In October 2015, control bronchoscopy failed to reveal an MDR *A. baumannii* and *P. aeruginosa* infection. Therefore, the antibiotic therapy that had lasted for six months, was discontinued. A few control chest CT scans were done. The last CT in October 2015 showed complete resolution of the abscess (Fig 1C). One year after the surgery, in March 2016, the patient was in a good condition with satisfactory lung function (FEV_1_ of 64.8%), receiving standard therapy for CF patients after LT (Fig 1D).

## DISCUSSION

Improvements in the treatment of CF have delayed but not stopped the disease progression. Due to significantly extended survival and improved quality of life, LT has become a viable treatment option in patients with CF. The most common and serious complications after LT in these patients are graft rejection and infection (non-cytomegalovirus). In addition to pneumonia, which is the most common type of infection, other infections include empyema, sepsis, and wound infection [1]. One of the less often complications is lung abscess. 

LT recipients, especially those with CF [2, 3], are at increased risk for infectious complications due to the high level of immunosuppression required to prevent rejection, adverse effects of transplantation on local pulmonary host defense (reduced mucociliary clearance, decreased cough), and constant environmental contact allowing pathogens direct access into the allograft. Furthermore, these patients remain at increased risk for infection because the native upper airways and sinuses retain the chloride channel defect and remain persistently colonized with the pathogens that were present pre-transplantation.

Infections generally occur in a predictable pattern of three stages: the first month, second to sixth month, and after six months. During the first month, bacterial infections predominate and are typically caused by hospital-acquired bacteria. This categorization is not absolute and infections may be distributed among alternative periods.

Patients with CF are more likely to be colonized with nosocomial MDRs, especially with *P. aeruginosa* and *Burkholderia cepacia* [4] among other pathogens, which are associated with the length of intensive care unit (ICU) stay and previous exposure to broad-spectrum antibiotics.

Lung abscess caused by MDR *A. baumannii *is a complication of LT that has not been reported before. There is no study that can tell us which bacteria predominate in LT recipients with CF. There are some case reports, none of them describing abscess as a complication in LT recipients caused by MDR *A. baumannii*. So far, only one case of disseminated B. gladioli infection was described characterized by bacteremia, necrotizing pneumonia, lung abscess and empyema in a patient with CF following LT. Due to misidentified microorganism at the start of the treatment, the outcome was lethal [5]. Therefore, it is of utmost importance to identify the causative microorganism to determine its significance and pathogenicity. This depends on multiple factors including the type of species isolated, colony count, and clinical manifestations.

To the best of our knowledge, there are no reports of prolonged antibiotic therapy in a patient suffering from lung abscess caused by MDR *A. baumannii* after LT recipient with CF.

No treatment recommendation has been issued by major societies specifically for treating lung abscess as an early complication in LT recipients with CF caused by the MDR *A. baumannii.* In general, the antibiotic treatment of lung abscess is guided by the microbiology sensitivity report. Although duration of therapy is not well established, most clinicians generally prescribe antibiotics for 4–6 weeks. Lung abscess may necessitate drainage with invasive techniques (percutaneous, endoscopic or surgical) along with either intravenously or inhalatory antibiotic treatment. We believe in LT recipient with CF, it is advisable to combine administration of iv antibiotics (guided by the microbiology sensitivity report) and percutaneous catheter drainage of the abscess (in case of large lesions) with inhalatory antibiotic therapy.

Antibiotic treatment should be prolonged until the chest radiograph has shown resolution of the abscess (the treatment may then take for several months). With evaluation and inclining of patient’s respiratory status, the patient may be treated on an outpatient basis for completion of long-term therapy.

There are no guidelines for postoperative management of bacterial infections in LT patients, resulting in significant variability among centers concerning antimicrobial prophylaxis, diagnostic strategies and therapeutic management. A detailed knowledge of local epidemiology is considered to be essential to ensure effective management of infections, particularly in the ICU. This task is becoming increasingly challenging with the emergence of MDR microorganisms. The goal of LT in all patients is to prolong survival and to improve the quality of life; therefore, it is advisable to have an effective strategy in dealing with complications of LT, primarily post-transplantation infections. Here, a case of successful treatment of rare complication (lung abscess) has been presented. With long-term antibiotic regimen we succeeded to eliminate infection and cure the patient.

Since no guidelines or recommendations on antibiotic regimen duration exist, this case report could help direct the clinicians when treating rare complications of LT such as lung abscess in patients with CF.
